# Supplementary material for: Cumulative route improvements spontaneously emerge in artificial navigators even in the absence of sophisticated communication or thought
Source: PLoS Biol. 2024 Jun 6;22(6):e3002644. doi: 10.1371/journal.pbio.3002644 (PMC11156315; doi:10.1371/journal.pbio.3002644)
Supplement: S1 File — A single PDF file is provided with figures that offer additional information. Fig A shows measures of efficiency as a function of the wgoal, wsocial, and wmemory components. Fig B shows the relationships between component weights and efficiency outcomes. Fig C shows example journeys and the landmarks that individuals used across generations. Fig D shows histograms of final efficiency and the intergenerational changes in efficiency for each condition. (PDF) [file pbio.3002644.s001.pdf]

SUPPORTING INFORMATION

Cumulative route improvements spontaneously emerge in artificial navigators even in the absence of sophisticated communication or thought

Edwin S. Dalmaijer [1]

Affiliation

[1] School of Psychological Science, University of Bristol, United Kingdom

Contact details

Dr Edwin Dalmaijer, University of Bristol, School of Psychological Science, 12a Priory Road, Bristol, BS8 1TU, United Kingdom. Email: edwin.dalmaijer@bristol.ac.uk

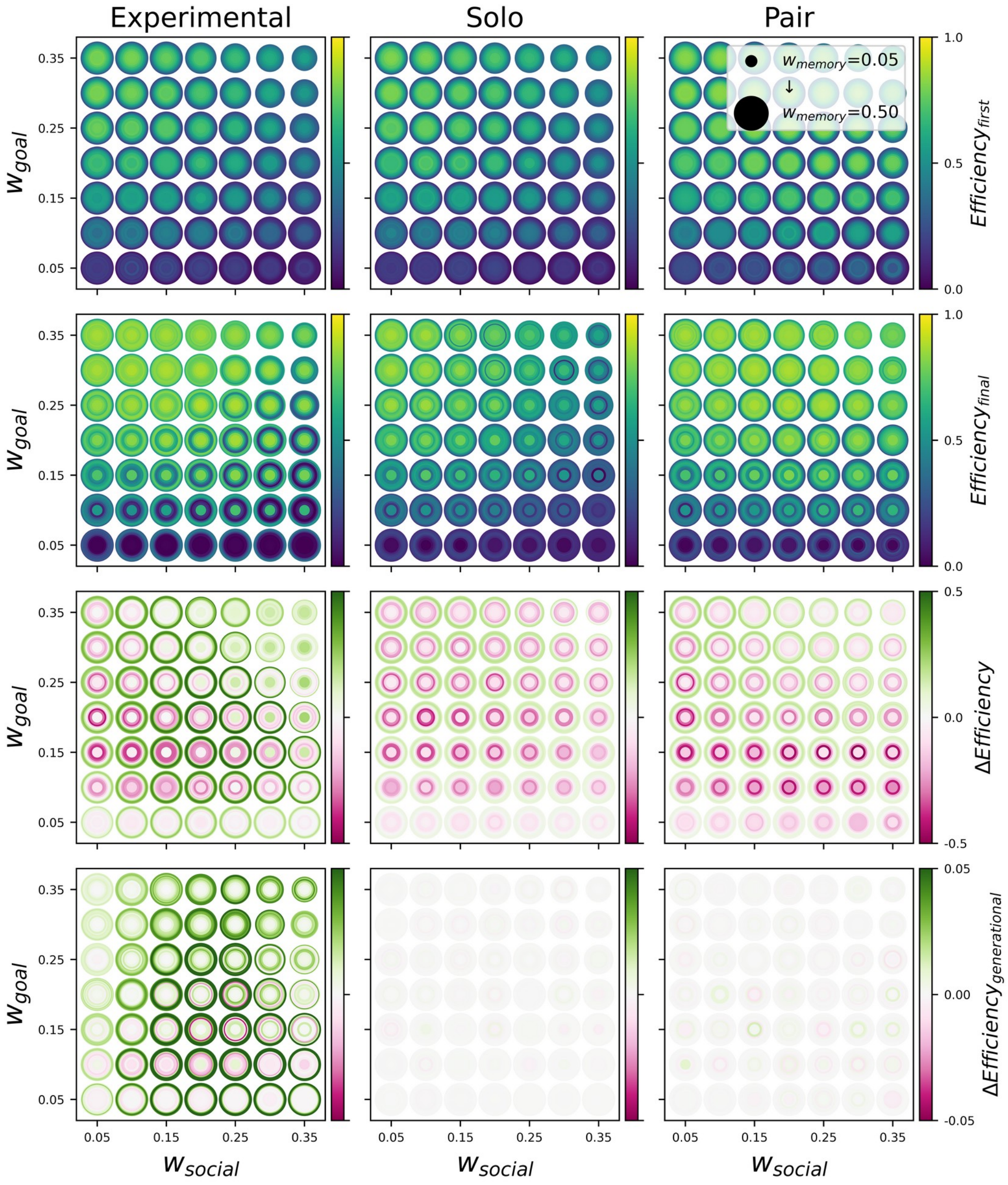

Figure A *– Each panel shows a measure of route efficiency as a function of w_{goal} (upwards on the y-axis), w_{social} (rightward on the x-axis), and w_{memory} (outwards on the disks). The first row shows the efficiency of agents' first journeys, with lighter colours indicating higher route efficiency. The second row shows the efficiency for agents' final journey (after 5 generations with 12 journeys each), again with lighter colours indicating better route efficiency. The third row shows the increase (green) or decrease (pink) in efficiency between first and final journey. The fourth row shows the average increase (green) or decrease (pink) between consecutive generations. Efficiency was computed as route length divided by Cartesian distance between start and goal. The data and code required to generate this Figure can be found on Zenodo: https://doi.org/10.5281/zenodo.6944185 (data) and https://doi.org/10.5281/zenodo.10997495 (code).*

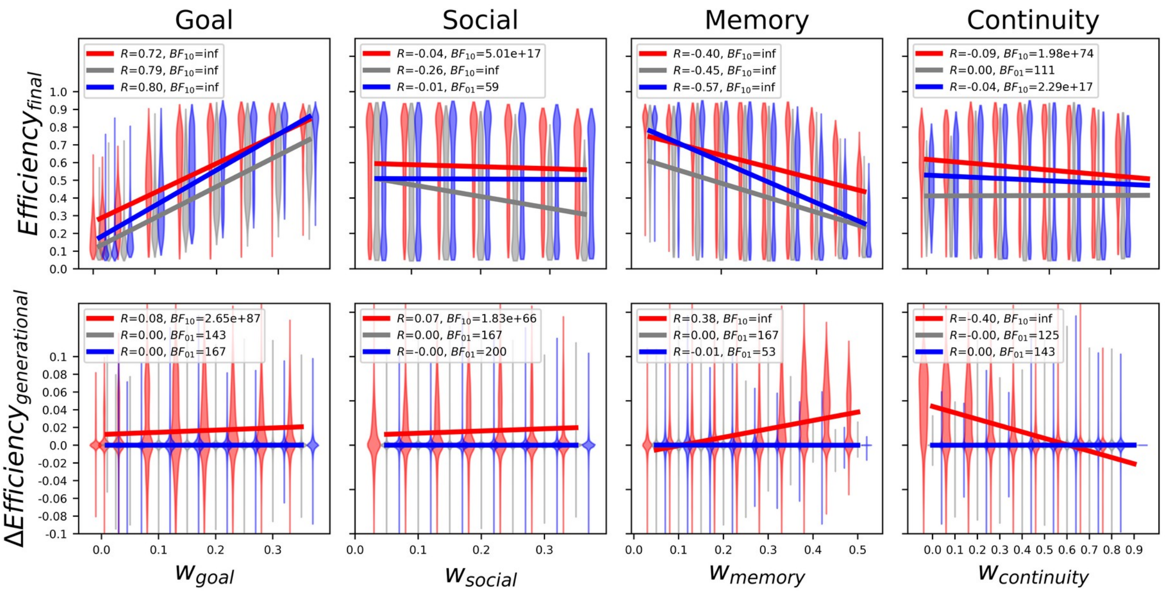

***Figure B** – Each panel shows the relationship between navigation component weights (w_{goal}, w_{social}, w_{memory}, and $w_{continuity}$; x-axis) and efficiency outcomes (top row: maximum efficiency in the final generation; bottom row: average inter-generational improvement). Collapsed across all simulations, distributions of outcomes are presented for each weight. Final efficiency is positively correlated with w_{goal} for all experimental conditions, and negatively with w_{social} (primarily for the solo condition, and not in the pair condition), w_{memory}, and $w_{continuity}$ (but not in the solo condition). Only in the experimental condition is inter-generational improvement in efficiency positively correlated with w_{goal}, w_{social}, and w_{memory} ; and negatively with $w_{continuity}$. The data and code required to generate this Figure can be found on Zenodo: https://doi.org/10.5281/zenodo.6944185 (data) and https://doi.org/10.5281/zenodo.10997495 (code).*

***Figure C** (next page) – This figure shows an example of a single complete run through 5 generations with 12 journeys each; with w_{goal}=0.20, w_{social}=0.15, w_{memory}=0.40, and $w_{continuity}$=0.25 for all conditions. The top row shows the route efficiency for each path, and the corresponding paths appear below. Columns indicate consecutive generations, and lighter lines indicate earlier journeys. Solid lines show one agent, and dotted lines the other. In generations 2-5 in the experimental condition, the solid is for the experienced agent, and dotted for the naive. Black dots indicate route landmarks memorised by the one agent, and white dots for the other agent (grey overlap if they are the same). Crucially, only in the experimental condition, landmarks slowly converge towards the optimal route from start (top right) to goal (bottom left). The data and code required to generate this Figure can be found on Zenodo: https://doi.org/10.5281/zenodo.6944185 (data) and https://doi.org/10.5281/zenodo.10997495 (code).*

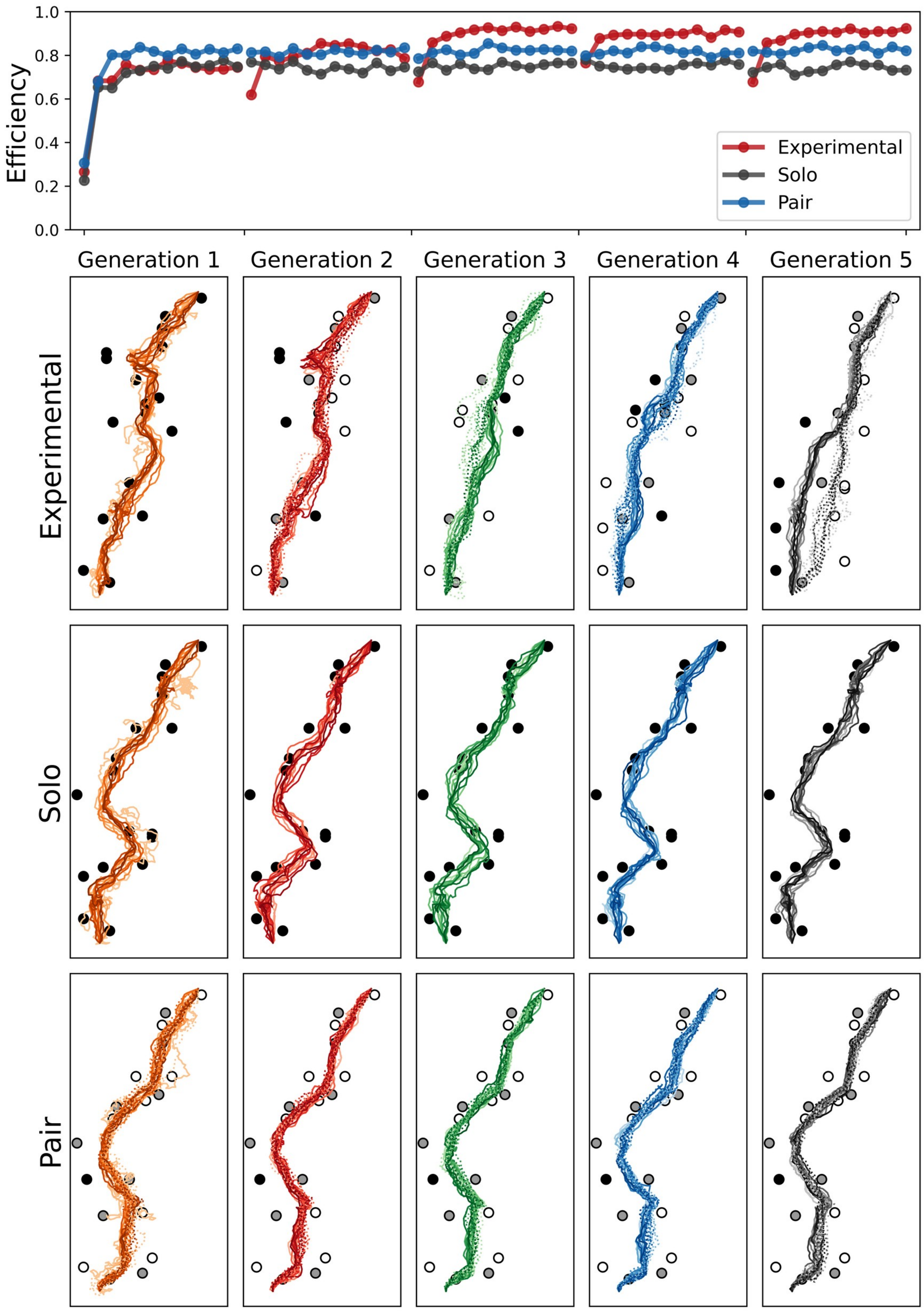

***Figure C** – Legend on previous page.*

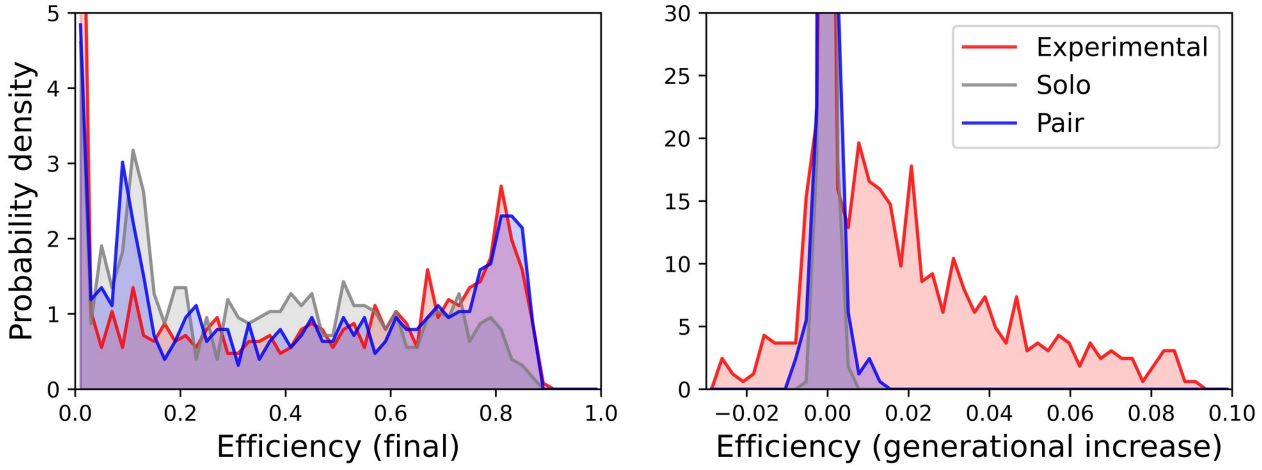

Figure D *– Histograms of the final-path efficiency (left column) and the mean inter-generational increase in efficiency (right column). In the experimental condition (red), a naive agent replaced an experienced one in each generation; in the solo condition (grey), a single agent made all journeys without generational turnover; and in the pair condition (blue), two agents journeyed together without turnover. The histograms were computed over all unique combinations of parameters, each represented as the mean over 50 independent runs. The experimental condition shows several parameter combinations with subtly higher final efficiency, and many for which inter-generational increases in efficiency are relatively high. The data and code required to generate this Figure can be found on Zenodo: https://doi.org/10.5281/zenodo.6944185 (data) and https://doi.org/10.5281/zenodo.10997495 (code).*
