# Supplementary material for: Cumulative route improvements spontaneously emerge in artificial navigators even in the absence of sophisticated communication or thought
Source: PLoS Biol. 2024 Jun 6;22(6):e3002644. doi: 10.1371/journal.pbio.3002644 (PMC11156315; doi:10.1371/journal.pbio.3002644)
Supplement: Attachment — Submitted filename: Dalmaijer_response_letter_cumulative_culture.pdf [file pbio.3002644.s002.pdf]

25 February 2024,

Dear editor and reviewers,

Many thanks for your thorough and constructive reviews.

Please find enclosed the revised version of my manuscript “Cumulative culture spontaneously emerges in social navigators with imprecise memory”. It has been edited to incorporate your suggestions, and I think this has greatly improved the manuscript. I am very grateful for your input.

In addition to the updated manuscript, I have included a point-by-point response with references to what changed in the manuscript. I hope will make it easier for you to review the revised version.

Many thanks for your continued consideration.

Yours sincerely,
Edwin Dalmaijer

Reviewer #1:

Before offering a point-by-point response, I just wanted to express my gratitude to the reviewer. I greatly appreciated the critical and constructive comments, and particularly so given that the reviewer took the time to build several models to compare against findings.

Major points:

1. The introduction is far too short. I'm all for getting straight to the point, but the current manuscript does so in such a hurry that it skips over a lot of context that would help get across why the reader should be interested in this work. I'm not going to tell the author how to write an introduction (I assume they are well versed in this), but as one example, it seems pertinent to cover a few other theories about what cognition supports cumulative culture and why those might not be the whole picture. The introduction should be at least double its current length, and could comfortably be much longer still.

ANSWER: The brevity of the introduction is a consequence of the "Short Report" format, but I also acknowledge that I was a little too enthusiastic to get to the point. In this revision, I have added several paragraphs to the Introduction that aim to provide a better overview of the literature on cumulative cultural evolution, the open debates, and how this paper relates.

As a consequence, the Introduction and Discussion have indeed about doubled.

2. Introduction paragraph 6. The spread (i.e. precision) parameters are fixed according to an analysis, but there is no reporting of the uncertainty in these estimates. Measures of such uncertainty should be provided. Even better would be a sensitivity analysis - does cumulative culture depend on the specific values used?

ANSWER: This is an excellent suggestion, and the results of such a sensitivity analysis are now included in Table 2. These findings are summarised in the manuscript in this way:

"Wider navigation component distributions reduced the inter-generational increase in efficiency, and a wider goal component even prevented agents from completing their routes. Narrower distributions effected less change, although a more precise goal component did subtly reduce inter-generational efficiency increases. The pattern of cumulative culture in the experimental but not in the pair and solo control conditions was apparent throughout."

3. Results regarding lesions of the goal directed component: "If they found the goal, this resulted in low-efficiency paths with ample room for inter-generational improvement. Indeed, inter-generational efficiency improvement still occurred in the experimental condition, but not in pair or solo controls. Hence, goal direction was not necessary for cumulative culture."

I found this result to be quite surprising, so surprising I think it must be incorrect. I've even built a few simple models to reproduce it while reviewing the manuscript and cannot do so. The issue is that that without the goal directed component I cannot see how there can be any means by which efficiency can ratchet upwards. For instance, suppose one pigeon completes a random walk, and they are then paired with another. The new pigeon will learn from the experienced pigeon, and will superimpose its own random walk on top, but this should be just as likely to worsen the route as to improve it. At first, I wondered if the information was coming from failed routes (those that never reach the goal) being discarded, but the manuscript makes clear this is not the case. Another option is pigeons are initialized facing in

the right direction on their first run (making the random walks not truly random). Or perhaps, this is an artefact of the efficiency measure: the initial random walks are at such low efficiency that the only way is up. But maybe I am wrong - if so the author needs to really dig into what is going on here and where the information is coming from that enables the population of achieve progressively higher efficiency.

ANSWER: This was a fascinating rabbit hole, and before we dive in, I'd like to offer the short summary: the reviewer is entirely correct. This result was artificial, and when done properly, agents in a no-goal control simply do not reach the goal, and agents in a no-goal-after-the-first-generation control do reach the goal but show no inter-generational improvement in efficiency.

What follows is an explanation of why there was accidental inter-generational improvement in the prior attempt. This is a deep dive, and since the issue is solved, I include it here only for the sake of satisfying curiosity. If one is uninterested or short on time, one could simply skip to "IMPROVEMENTS IN THE REVISED MANUSCRIPT".

WHY WAS THERE INTER-GENERATIONAL IMPROVEMENT IN NO-GOAL CONTROL?

My main mistake here was to let routes continue for 14800 steps, while the minimum path from start to goal spanned just 122 steps. As described in the paper, agents with p_{goal}=0 engaged in a random walk that often ended up unfinished, but with 14800 steps chances were high that at least some random walks accidentally ended up at the goal. This accidental goal-finding was also helped by my setting the initial heading of each agent towards the goal.

While the above explains why no-goal control birds ended up finding the goal, it does not in itself explain why they also improved. This was a byproduct of the limited number of landmarks across a route. The exceedingly long random walk in the first journey was simplified to a path across 10 landmarks, thereby replacing the meandering nature of the original path with an oddly shaped but shorter route. This was the first cause of improvement, and could boost efficiency from e.g. 0.01 to 0.23 (most finished no-goal control runs started on 1-3% and ended at 10-30% within the first generation).

While the above explains why no-goal control birds improve within the first generation, it does not explain why they improve between generations. As the reviewer rightfully notes, regression to the goal cannot exist when p_{goal}=0, and thus cannot be the explanation for intergenerational efficiency improvements. Instead, the explanation seems to be in another interaction between naive and experienced agent. Within each generation, a naive agent undergoes the previously described process of random-walking without landmarks until accidentally hitting the target, and then following the landmarks to improve upon the initial meandering pathway. However, from the second generation onwards, the naive agent is no longer truly random-walking. Instead, they are also following the experienced agent, who is bound for the target. Thus, the naive agent's initial path is biased towards being more goal-oriented than a completely random path. Ultimately, this means there is a chance for subtle improvement between each generation.

The control data supports this: the efficiency range of the naive agent is invariably wider than that of the experienced agent, with a minimum efficiency that is almost always (39/40 experimental generations) worse than the experienced agent's average efficiency, but a maximum efficiency that tends towards being higher.

Further, when both p_{goal} and p_{social} are set to 0, no inter-generational improvement can be found. This suggests that the social proximity is indeed necessary for the no-goal control to still show

some inter-generational benefit when their number of memorised route landmarks is artificially limited.

WHY WAS THERE NO INTER-GENERATIONAL IMPROVEMENT IN OTHER CONTROL CONDITIONS?

The reason this is not present in the pair condition, is that paired agents do not change their landmarks throughout all "generations", and hence no accidental improvement is possible within any generations after the first.

The reason this is not present in the no-social control condition, is because the agents simply do not follow each other. Instead, each naive agent undergoes the exact same process of accidental efficiency improvement due to the original meandering (but now with goal-direction) being replaced with a more direct path along the landmarks. As this is equally likely to be better or worse, on average there is no benefit.

The reason this is not present in the no-memory control, is that the landmarks have no impact on the path. Thus, every single path is another random walk (or somewhat goal-directed, if $p_{goal}>1$), which prevents accidental improvement within a generation.

IMPROVEMENTS IN THE REVISED MANUSCRIPT

While I think it's highly defensible that all agents start with the same initial heading, as it avoids random variance in the original heading potentially biasing results, I have now adjusted the start heading to be 0 (north) instead of the relative heading towards the goal.

Further, I have reduced the maximum number of steps by a factor of 5. For reference, the Sasaki & Biro experiment occurred over a distance of 8.5 km, which was analogous to 122 steps in the reviewed manuscript, but I set the maximum route duration to 14800 steps. This did not account for fatigue, which agents do not suffer from, but real birds do. Specifically, pigeons show a marked increase in reactive oxygen metabolites and a decrease in serum antioxidant capacity (Constantini, Dell'Ariccia, & Lipp, 2008, Journal of Experimental Biology), and they also show continuously increasing concentrations of uric acid and other metabolites (Schwilch, Jenni, & Jenni-Eiermann, 1996, Journal of Comparative Physiology B) after flights that spanned 200 km and lasted 5 hours. This corresponds to about 2857 steps in my simulations, i.e. about 20% of the original maximum number of 14800. In relative terms, the previous maximum was 122 times the start-goal distance, and the new maximum is 23.5 times that.

In sum, agents are now considered lost if their trajectory exceeds 24 times the start-goal distance.

This is now captured in the manuscript:

"Agents travelled 1 distance unit per 1 time unit, attempting to find a fixed goal from a fixed starting point that were 104 units apart. The maximum distance agents were allowed to travel was 2506 units. Compared to the map used by pigeons in Sasaki and Biro's study (2017), this is equivalent to a flight of 200 km and ~5 hours. This cut-off was chosen because pigeons would have suffered continuously increasing concentrations of uric acid and other metabolites (Schwilch et al., 1996), and a marked increase in reactive oxygen metabolites and decrease in serum antioxidant capacity (Costantini et al., 2008)." (section "Experimental Design")

The updated control results are also included in Table 1, and the section "Control experiments with lesioned agents".

4. Discussion. Like the introduction the discussion is really short. There needs to be more of an effort to expand on how this work contributes to and interacts with other parts of the literature.

ANSWER: This was also due to this being a Short Report submission, and again I had overdone the brevity. I have now expanded the Discussion to address how my findings contribute and interact with work on both humans and non-human animals.

Minor points:

1. Introduction paragraph 3. There are semicolons between the first three rules, but a period (full stop) between the 3rd and 4th. Any reason for this inconsistency?

ANSWER: The reason for this inconsistency is mostly that I see the first three rules as core to the agents' behaviour, and the fourth (continuity) as a simple trick to avoid erratic patterns. However, I do see that the semicolons made for an awfully long sentence, so I have now separated the rules with full stops.

2. Introduction paragraph 3. Continuity is introduced but not defined, leaving the reader to ponder what it means.

ANSWER: This is now further clarified:

"The fourth is continuity, a tendency to continue along the current heading to avoid implausibly erratic patterns."

3. Introduction paragraph 4. Von Mises distributions are introduced but not explained. I had to look them up on Wikipedia. It would be perfectly easy to explain they are basically circular normal distributions. It is important to explain this because these distributions are a core part of the model.

ANSWER: This is now clarified directly after Von Mises distributions are first mentioned:

"These are akin to normal distributions, but they are circular, so that the tails wrap around."

4. Introduction paragraph 4. The existence of b_goal needs more justification. As it is, it is magic knowledge of where the goal is. The author needs to justify why it is reasonable to simulate agents with this kind of knowledge. A more detailed discussion of solar/magnetic compasses would be a good step.

ANSWER: I have elaborated upon the solar/magnetic compass in the introduction:

"The first is goal direction, akin to birds' solar (Kramer, 1952) and magnetic compasses (Keeton, 1974) that allow them to orient towards their home even from unfamiliar release sites unless under total overcast with disorienting magnets glued to their head (Keeton, 1971)."

The additional reference (Keeton, 1971) has beautiful illustrations of birds orienting towards home from both familiar and unfamiliar release sites (see distributions around the dashed home arrow in figures 4-6 and 7-9).

I do apologise for still being rather brief on the subject here; the Short Report format does not allow for many words, and I hope that curious readers will turn to Keeton (1974) in particular. In the Methods, where more words are allowed, I have included the following elaboration:

"The first rule was goal direction. The centre of this distribution was the bearing towards the goal bgoal, its precision parameter was κgoal , and its weight wgoal. The bearing was computed from the coordinates of the goal (xgoal,ygoal) and agent at time t (xt,yt) (Equation 2). The purpose of this rule was to orient agents towards the goal, just like pigeons can orient homewards upon being released from unfamiliar sites. This ability likely depends on the sun, as starlings and pigeons can learn to use light and time-of-day to orient towards rewards (Kramer, 1952), and pigeons orient homeward when the sun is visible (Keeton, 1971). They can even do so when it is overcast, but their initial orientation becomes more random when magnets are glued to the back of their heads (Keeton, 1971), suggesting that pigeons also use an internal compass. For more comprehensive overviews, see (Keeton, 1974) and (Beason & Wiltschko, 2015)." (section "Artificial navigator model)

5. Introduction paragraph 4, and throughout. The k parameter is defined as a spread, but it's not, it's the reciprocal of a spread, more intuitively called concentration or precision.

ANSWER: This is an excellent suggestion! I've now reworded "spread parameter" to "precision parameter" throughout the manuscript.

6. Introduction paragraph 4. The author states that uncertainty in the location of the goal is greater than uncertainty in the location of the next landmark. Is this an assumption of the model or an empirically documented fact? It needs greater explication and justification.

ANSWER: I have further clarified this in the Introduction:

"For example, there is uncertainty about where the (solar/magnetic compass) goal is (Keeton, 1974; Kramer, 1952), whereas pigeon visual acuity is good enough (Blough, 1971; Hahmann & Güntürkün, 1993) to identify nearby visual landmarks along a well-memorised route (although they are not always used, (Beason & Wiltschko, 2015))."

The notion of visual landmarks being used to guide avian navigation is not without debate. For example, while Keeton (1974) recognises birds can visually identify landmarks, they are quite dismissive about birds actually using them in navigation. A recent review on the topic presents a similar view (Beason & Wiltschko, 2015). Although I find the cited work on pigeon navigation by landmarks (Lau et al., 2006; Mann et al., 2011) quite convincing, I'm not well-placed to persuasively argue for any side in this debate. This is also not the venue for it, because ultimately, the most important question is whether the decision to model goal-direction as less precise that memory in the artificial navigator model impacts the findings. It is thus entirely fair that the reviewer would like to see the effects of this decision.

I have run an additional set of control experiments in which the precision parameters of the goal and memory components are independently varied, and the effects on inter-generational improvements in efficiency are measured. In short, the precision parameters do impact efficiency, but the pattern of inter-generational transmission in the experimental but not the pair or solo conditions is retained. (Results reported in Table 2.)

7. Introduction paragraph 5, and throughout. It is never really stated that the experimental condition also involves pairs. Rather, turnover is mentioned, but not the group size.

ANSWER: This has now been made explicit:

“The experimental condition also involved pairs, but a naive replaced an experienced agent every 12 journeys.”

8. Introduction paragraph 6. The term "runs" needs to be more clearly defined.

ANSWER: The term “runs” is now replaced by “repetitions” to make it clear that it concerns a repetition of the same set of parameters.

9. Methods. Is it plausible that b_other pulls you towards the other pigeons projected location? At high enough weights this will cause pigeon collisions. My recollection of the flocking literature is that individuals align their headings with those of nearby individuals, and not that they explicitly fly at them.

ANSWER: This is a fair point, and indeed social weights towards 1 could result in collision or in agents circling each other. This approach is a somewhat awkward compromise that accounts for practical conditions: at low w_{social} values, agents aren’t necessarily expected to travel particularly close together. When further away, aligning headings does not work to move agents in the same direction: they would instead start to fly in parallel and thus never get closer! However, aligning towards each other’s predicted locations would still move agents towards each other.

As the reviewer noted, birds align headings with those who are nearby, but not necessarily those who are further away. I have thus implemented alignment at close range, while still allowing agents to converge towards each other at greater distances.

The approach is now as follows: agents’ social heading is now determined by alignment at close proximity, and convergence at less close proximity. The final social heading is a weighted circular average of the other agent’s heading and the other agent’s predicted location. The weight is determined using a normal distribution around 0.5 and a standard deviation of 0.1, which is equivalent to ~30 metres in the pigeon experiment by Sasaki & Biro.

The 30 meters figure is based on an observation by Delius (1963), who notes that skylarks can recognise each other’s sex at 50 metres, and can actively recognise individuals at 30 metres. (“[…] wie aus ihrem Verhalten zu entnehmen war, konnten sie schon aus 50 m Entfernung die Geschlechter gut bestimmen.” and “Wie ihr Verhalten zeigte, erkannten sie ihren Partner optisch sehr gut auf 30 m Entfernung, akustisch vielleicht noch weiter.”, page 314)

The passage in the manuscript now reads:

“The second rule was social proximity. This distribution is a weighted composite of a Von Mises distribution for social convergence that is centred on the bearing towards another agent’s estimated future position $\hat{b}$other, and another Von Mises distribution for social alignment that is centred on another agent’s current relative heading. The alignment of headings between agents at close proximity is a crucial part of flocking behaviour (Reynolds, 1987), but at larger distances agents need to converge rather than align to achieve social proximity. Samples drawn from the convergence distribution were weighted with proportion p and those drawn from the alignment distribution with (1-p). Proportion p was drawn from a cumulative normal distribution with mean 0.5 and standard deviation 0.1, which is equivalent to a distance of 30 metres, at which pigeons are estimated to be able to recognise individuals (Delius, 1963). Both composite distributions have precision parameter κsocial, and the combined distribution has weight wsocial.

Bearings towards other agents were computed from an agent's position at time t, (xt,yt), and other agent j's expected position at time t+1 (Equation 3). The expected position of agent j at time t+1 was estimated on the basis of their velocity v (which was kept constant) and their heading hj,t at time t (Equation 4)." (section "Artificial navigator model")

10. Methods. Route memory. It is important to make clear here that the landmarks are therefore unique to each agent. This is not intuitive, as in the real-world landmarks are typically conspicuous features (a lake, or tower etc) and so agents cannot freely choose their locations. But here, part of the reason that efficiency can improve is that each agent generates its own landmarks from a continuous landscape allowing for gradual improvements. This sort of thing would be suitable for an expanded discussion.

ANSWER: This is an excellent point, and I appreciate the chance to elaborate on this further. My decision to implement landmarks on this is based on Lau et al. (2006), who show that potential landmarks along pigeon flight routes seem to overlap with areas identified from aerial photos with edge detection algorithms, and Mann et al. (2011) who detect landmarks using statistical models and pigeon flight data.

These studies shows that the reviewer is correct: real-world landmarks are based on conspicuous features and thus agents cannot completely freely choose. It also shows that my assumption could be considered a close-enough implementation, because while the number of possible landmarks was not infinite (like under my assumption), it wasn't particularly sparse either: a high number of potential landmarks was found using edge detection alone.

The ideal way to settle this was to empirically test it. I implemented a landscape with sparse landmarks that agents remember upon passing close to them. The results are closely aligned with the previous results, which suggests that my simplification of continuous landmark locations was a good approximation of actual fixed landmarks.

To be clear: the findings reported in the revision are on the basis of the new fixed-landmark algorithm. This has been updated in the Methods section:

"Across the map of 200 by 130 units, 6500 landmarks were spread. This aligns with landmark detection using pigeon flight routes (Mann et al., 2011) and edge detection in aerial photography (Lau et al., 2006)." (section "Artificial navigator model")

11. Methods. Experimental design. It is not clear how the choice of speed (70 units per 1 time unit) affects the results. This needs an expanded justification (or clarification of why it doesn't matter). If it does matter, a sensitivity check would be helpful.

ANSWER: My choice of speed was not elegant (roughly based on Sasaki & Biro's map), and I have redone the simulations with a step of 1 unit of distance per 1 unit of time. The results (which now appear in the revision) closely align with the findings from my original submission, so it seems the choice of speed is not particularly important.

12. Methods. Experimental design, Weight parameters. How are all these weight parameters possible? Many combinations would exceed a sum of 1? In the supplementary figures it looks like only valid combinations are considered, but this needs to be stated.

ANSWER: Only combinations adding up to 1 were included, which is now clarified in that section of the Methods:

"No combinations with weight sums over 1 were included, and for $w_{continuity}$ made up the difference for all weight sums under 1."

This was already reflected in the listed numbers of unique combinations, which thus remain unchanged.

13. Methods, Data reduction. How many samples (and what %) were discarded for either GPS glitches or excessive distance from the route?

ANSWER: This has now been clarified in the manuscript: "Out of 2176 files in the original dataset, 6 were excluded for straying too far off course, and 45 for not reaching the goal." (section: "Data reduction and statistics: pigeons")

Note that each of these files contains data from a single bird, so one would have expected 600 from the solo condition (10 repetitions of 60 flights by 1 bird each), 1200 in the pair condition (10 repetitions of 60 flights by 2 birds each), and 120 (10 repetitions of 12 solo flights in the first generation) plus 960 (10 repetitions of 4 generations of 12 flights for 2 birds each) from the experimental condition, for a total of 2880. There are thus 704 missing files (2880 flights – 2176 files) in the original dataset. Sasaki & Biro have already addressed this discrepancy in their paper:

"One experimental pair in the fourth generation (and the subsequent pair in the fifth generation) and another in the fifth generation were excluded from analysis because the birds split up during all 12 of their flights (splitting was defined as individuals released in a pair becoming separated by more than 150 m at any point during flight 10). Additionally, one bird in the solo control group did not return home on its thirteenth release. In the fixed pair control group, three birds belonging to different pairs did not return home on their first release. We also excluded a pair that split up in more than 90% of their total flights. Thus, the sample size for the experimental group was 9 in the fourth generation and 8 in the fifth generation, for the solo control group it was 9 from the thirteenth release onwards and for the fixed pair control group it was 6 from the first release."

14. Methods, Data reduction. There is a reference to "the original paper", but which paper is this? What exactly is the subtle deviation? How can we be sure it doesn't matter?

ANSWER: The reviewer refers to the passage:

"No further exclusions were done, and incomplete flights were not imputed. This method subtly deviates from the original paper, but the pattern of results is the same (Figure 2, bottom)."

Which has now been rewritten to:

"Sasaki & Biro (2017) also imputed several early incomplete flights with direct-to-home trajectories, which was not done here to avoid fitting models to imputed data, but the pattern of results matches nevertheless (Figure 2)."

This clarifies what the "original paper" is, and makes it explicit that the "subtle deviation" is the lack of imputation of incomplete paths that go directly home. I think this is sensible, because my goal is to fit a model to real data, whereas Sasaki & Biro (2017) were trying to salvage trials with incomplete paths. Note that they wrote the following about these imputations, which they stress did not impact their conclusions:

"In total, 1,080 flights were recorded in the experimental group, 552 in the solo control group and 840 in the pair control group. Of these, 59 (5.4%), 22 (3.9%) and 31 (3.6%), respectively, failed to provide full track data due to GPS device failure. These flights are therefore missing from the analysis. In addition, on 22, 5 and 4 occasions, respectively, birds took longer than the device's battery life to return home, and thus we obtained only partial GPS tracks from these flights. For our analysis, we assumed that these birds flew directly home from the location where they were at the time the GPS battery ran out; we therefore underestimated their routes as their actual routes were most likely considerably longer. In all three groups, these occasions were confined only to the first 3–4 releases of any given bird (that is, within generations in the case of the experimental group). Because we did not use data from early flights in our comparisons of route efficiency and route similarity between groups, any bias we might have introduced into these flights by estimating their missing portions had no effect on our overall conclusions." (Sasaki & Biro, 2017, page 5)

15. Methods, Data reduction. Why is efficiency computed from the best of 12 journeys? Why not the average across all 12? Does this matter?

ANSWER: The approach used by Sasaki & Biro (2017) was to use the last journey, which they assumed was the best, but this isn't necessarily true. Within a generation, an asymptote to efficiency is reached, and after this paths randomly fluctuate around this asymptote. This can clearly be seen in pigeon data from the pair condition (Figure 2, bottom panel; and Sasaki & Biro (2017)'s Figure 2, top panel). Hence, to capture the true "highest efficiency", one should take the best out of 12 rather than the last out of 12.

The above distinction is not merely academic, but evident in empirical data: Sasaki & Biro (2017)'s efficiency dips in flights 9, 10, and 12 in generation 5 for the solo and pair condition, but not for the experimental condition. This is presumably due to random fluctuations (at least I don't see any other explanation), which means that a direct comparison between these conditions would be biased towards the experimental condition, and a best-out-of-12 approach seemed less biased.

The reviewer asks about averaging all 12 journeys. This would also include the first several journeys, which by definition should be worse: landmark memory during these journeys is still improving, and thus efficiency is worse. This is true for all experimental generations, but only for the first control "generation" (which are stable solo or paired individuals). Hence, averaging over all 12 journeys would be an unfair comparison.

16. Figures S1 and S2. These figures are pretty, but the nested circles render them almost incomprehensible. I'm not sure what the best solution is, but perhaps separating the plots by w_memory is the only option.

ANSWER: I have now added a figure that shows the correlation between each separate weight parameter and efficiency (both final and inter-generational improvement).

Reviewer #2:

This article, "Cumulative culture spontaneously emerges in social navigators with imprecise memory", describes an avian navigation model where agents follow just four rules. Navigators that were paired outperformed solo navigators, and when the composition of the pairs was varied rather than fixed, efficiency improved even further. Interestingly, both the experienced and naive individuals benefitted from each other, which was somewhat unexpected: one would not think (at least initially) that the naive individual would be of much benefit to the experienced one.

This model and the findings of this paper pose a provocative question to the field of non-human culture, where there is a lot of debate currently about this topic (see Whiten 2023 Physics of Life Reviews, and the slew of responses). I think there is a lot of value in papers like this, which challenge the prevailing lines of thought in the field, which has at times become stagnated, and has always been too anthropocentric for its own good. It's an interesting time for the field, with a lot of new findings emerging (or on the brink of doing so). So, I would be happy to see this article published, and I do think that PLOS Biology would be a good fit. However, I do have some comments for the authors to consider first.

I am admittedly not an expert in modelling, so cannot really comment on the exact ins and outs of the model itself - although I can't see any glaring issues with it, the parameters are quite thorough and the controls seem reasonable. Hopefully, the other reviewers will be able to comment more thoroughly on this. My comments will therefore focus mainly on the introduction and discussion, which are at present, very brief, and could use expansion (or, at least reference to certain points - I am aware this is a short report, so won't necessarily be able to include much depth, but at least referencing would be helpful).

ANSWER: I would like to thank the reviewer for their very kind words!

The original paper by Sasaki and Biro, which involved releasing pigeons (as singles or pairs, of either fixed or variable composition) is one of my favourites. While I am personally inclined to agree that Sasaki and Biro demonstrated elements of cumulative culture in their pigeons, the wider field tends to be more skeptical. For example, in the introduction, the authors state "innovations that meet core criteria for cumulative culture also occur in various other species" and cite Mesoudi and Thornton, 2018. Again, while I like the Mesoudi/Thornton criteria and believe these are more sensible than some of the others in the literature, these are just one set of potential criteria that may be used. The original Tomasello definition and criteria are still widely used, particularly in terms of increased 'complexity' (rather than efficiency, which is what the Sasaki paper shows). Many researchers would not accept that cumulative culture in any form has been shown in animals. At present, this paper doesn't touch on the existence of this debate at all and presents cumulative culture in pigeons as if it is a fact - which is a little "jarring" to read as someone from the field, even if I agree with it! I would strongly recommend the introduction and discussion be expanded to reference this (and then state that the Mesoudi/Thornton definition was actively picked over the others, explaining why). I don't think including this hurts the novelty of the paper or its conclusions at all: rather, it may actually strengthen them. Offshoots of the Tomasello definition/criteria often involve a requirement for imitation/teaching or other high fidelity copying, which this model would appear to disprove the need for.

ANSWER: The brevity of the original manuscript was inspired by this being a Short Report submission, but I nevertheless completely agree with the reviewer that crucial context was missing.

I have expanded the Introduction and Discussion to incorporate work on human cumulative cultural evolution.

The revision expands upon the Tomasello and Tennie arguments against non-human animals having any form of cumulative culture. While I lack the space to comprehensively address this issue, I do introduce it, and then point towards the recent Whiten (2022) target paper and its responses.

In addition, the Introduction now offers practical examples of cumulative culture in non-human animals. It then makes a clear distinction between Type I and Type II cumulative cultural evolution, and introduces the Mesoudi & Thornton in that context.

The Discussion has also been expanded to allow reflection on what the current findings add (including along the reviewer's suggested lines), on where they are applicable to human cumulative culture, and on where they align with empirical studies on animal culture.

Reviewer #3:

This manuscript presents an interesting simulation model based on the prior transmission chain experiment on homing pigeons by Sasaki and Biro. Simulations demonstrate how naive agents, while following experienced agents, are more likely to deviate towards the goal, biasing the pair towards more direct flight paths. Authors conclude that these cumulative benefits might arise from simple systems that seek social proximity and are able to forget prior information.

Overall I very much liked the paper. It is another exploration of how naive individuals may drive adaptive cultural change, in the behavioral context of flight routes. I have a few major concerns to bring up:

When these concerns are addressed, I do think it will be a very nice addition to the literature.

ANSWER: Many thanks to the reviewer for their positive appraisal and their constructive feedback!

1. The introduction is quite terse and misses an opportunity to frame this model better in the literature and highlight it's relevance outside of the context of pigeon flight. There have been other studies on the effect of naive individuals in different contexts. See comment #1 under introduction.

ANSWER: The brevity was a consequence of this being a Short Report format submission, but I do agree with the reviewer that I overdid it. The Introduction has been expanded to frame the model better within the literature, and the suggested studies on naive individuals have also been incorporated in the revision.

2. The results focus on the case where the experimental condition outperforms pair and solo, but the cool thing about this model is that it gives insight into when this wouldn't be the case. Author should highlight parameter constellations where the predictions don't match the experimental results. The "lesion" experiments begin to address this, but there must be cases where even without lesions the experimental condition under-performs.

ANSWER: I have now additionally highlighted where naive agents underperform in the experimental condition (see Figure 3 and its discussion in text). I have also added a new visualisation (Figure S2) that shows more general patterns in which parameters positively and negatively impact efficiency and inter-generational improvements in efficiency.

3. It seems like all individuals know the location of the goal. What happens when only the first generation knows the location of the goal? It's been a while since I've looked at the design of the original experiment, but this seems like it's an important point because it bakes in the likelihood of naive agents moving towards the goal more directly. The lesion experiment does not quite test this point, since all individuals were lesioned.

ANSWER: The reviewer's suggestion is a brilliant way to isolate the effect of goal-direction on cumulative culture without losing agents who go on random walks. I have thus implemented this in another lesion study, in which the first generation does have a functioning goal-orientation, but generations 2-5 do not.

The result is that while agents in this control condition now reach the goal successfully, no inter-generational increase in efficiency occurs. This underscores the importance of goal-direction for cumulative culture in artificial navigators, and helps to confirm that an experimental pair's

regression to the goal as inspired by naive agents is indeed what drives inter-generational efficiency improvements.

In the manuscript, the above results can be found in Table 1, in the column "Goal lesion (gen>1)".

(The reviewer raised this again later, in the context of another point. The above text is copied in my response to that later point, but supplemented with additional explanation. I do apologise for the repetition; it seemed prudent to have the full context in both cases.)

4. A final major point to think about is the relationship between weights in the calculation of heading h, particularly goal and social. I was wondering whether all weights should sum to 1 for the final decision, or whether each weight should vary independently. Maybe this is the case, but I couldn't find it in the methods, if so ignore this point but make it explicit in your initial description of methods. I guess the question is should the influence of one factor come at the cost of another factor? Also if they're allowed to vary independently, every case where w_goal=w_social is redundant?

ANSWER: The weights do indeed add up to one, and this is now made explicit in the Introduction:

"The artificial navigator model is a weighted mixture of Von Mises distributions Φ, with weights w that add up to 1 (Equation 1)."

This was already noted in the Methods section, but only once, so it was easy to miss:

"Weights were set at agent initialisation, and added up to 1." (section Artificial navigator model)

In the revision, it is now stressed again in the Experimental design section:

"No combinations with weight sums over 1 were included, and $w_{continuity}$ *made up the difference for all weight sums under 1."*

Abstract

1. **"…cumulative culture was attenuated when agents social proximity or route memory were lesioned, whereas eliminating goal direction only reduced efficiency". This sentence made me stumble and I had to reread it. Social proximity is an emergent property, wheres memory and goal direction are a parameterized characteristics. maybe rephrase so all 3 are parameterized characteristics, and split into two shorter sentences**

ANSWER: This sentence is now rephrased:

"In control experiments, cumulative culture was attenuated when agents' goal-direction, social alignment, or route memory were lesioned."

Introduction

- a side-note to authors, please include line numbers when submitting papers!

ANSWER: While I fully agree with the reviewer on this, I have been burned by submitting preprints and manuscripts in the past to venues that explicitly disallow them.

It seems PLOS Biology does recommend using them, so I have now included them in the revision. Apologies for the inconvenience of not having them previously!

1. pg 3 - the introduction is highly focused, which i like, but you miss an opportunity to frame the paper in a broader context of how naive individuals shape culture across behavioral contexts. See Chimento Alarcon & Aplin 2021 for foraging context, Warner 1988, 1989 for spawning resource choice context.

ANSWER: The submission is a Short Report, but I agree that I might have overdone the brevity. In response to the other reviewers, the introduction is now expanded to include more human cumulative culture work. However, I do appreciate the additional references provided by this reviewer, and I have incorporated them in the Discussion:

"My results also demonstrates a role for naive individuals in cumulative culture. This aligns with findings from bluehead wrasse, which use traditional mating sites (like paired agents stick to idiosyncratic routes), but adopt new sites upon population refresh (Warner, 1988, 1990). It also aligns with empirical work in great tits, in which population turnover drove cumulative improvements in efficiency due to new naive individuals conforming to efficient variants (Chimento et al., 2021)."

2. pg 5 include h -> to produce next heading h in journey…

ANSWER: Thanks for spotting this oversight! Now included.

3. Pg 5 - what is a "clean run"?

ANSWER: I have now rephrased this to "repetition", which I hope is clearer.

4. Pg 5 - 10 runs per parameter constellation is very low for a modeling paper. I know it won't change the general shape of results, but should be higher N

ANSWER: The model is quite consistent and the covered parameter space is large, so I kept the number of repetitions low to save time. (It doesn't help that I don't have ready access to a computing cluster, so the software runs on a decade-old computer I've had since my PhD!) Obviously, the reviewer is correct, and so I increased the number of repetitions to 50.

On a related note: It's good to keep in mind that within each of these repetitions, 60 journeys are made across 5 generations, so the actual N is rather large!

Results
5. Pg 6 - the reported means should include some measure of variance.

ANSWER: Fair suggestion! Standard errors of the mean are now reported alongside averages.

6. Pg 6 - why isn't continuity reported?

ANSWER: Because the weights add up to 1, continuity is the remainder. The reviewer is absolutely right that reporting all weights is much clearer, though, so all parameters are now reported.

7. Pg 6 - a bit confused what the "parameters closest to the final-route efficiency peak" means. How can parameters be close to a measured outcome?

ANSWER: This was an awkward section that has now been rewritten.

8. Pg 6 - In general, a bit more space could be dedicated to reasoning through the "why" of these results. The specific report numbers are less important/informative to the reader on their own, more important seems like the ratio between weights.

ANSWER: I have attempted to provide more context, and I think Figure S2 in particular will offer a more general overview of how each individual parameter impacts efficiency outcomes.

9. Pg 6 - Could also discuss when the experimental condition under-performed and why? This this equally as important

ANSWER: I have now additionally highlighted where naive agents underperform in the experimental condition (see Figure 3 and its discussion in text). I have also added a new visualisation (Figure S2) that shows more general patterns in which parameters positively and negatively impact efficiency and inter-generational improvements in efficiency.

10. Pg 8. Do naive individuals begin knowing where the goal is? If so, what happens if they don't have any information about the goal, and only the first generation of individuals knows the goal? This seems like it bakes in the result that naive individuals are more likely to head towards goal without route memory.

ANSWER: Just like pigeons, artificial navigators have a rough idea of where the goal is. This is reflected in the (wide) distribution towards the goal, and how much agents use this is determined by the w_{goal} *parameter. In the control experiments where goal-direction was lesioned, none of the agents knew where the goal was. (Instead of from a goal-centred distribution, headings were sampled from a uniform distribution.) As a consequence, they are forced into a random walk, and do not reach the goal within time.*

The reviewer's suggestion is a brilliant way to isolate the effect of goal-direction on cumulative culture without losing agents who go on random walks. I have thus implemented this in another lesion study, in which the first generation does have a functioning goal-orientation, but generations 2-5 do not.

The result is that while agents in this control condition now reach the goal successfully, no inter-generational increase in efficiency occurs. This underscores the importance of goal-direction for cumulative culture in artificial navigators, and helps to confirm that an experimental pair's regression to the goal as inspired by naive agents is indeed what drives inter-generational efficiency improvements.

In the manuscript, the above results can be found in Table 1, in the column "Goal lesion (gen>1)".

What the reviewer considers "baked in" is what I described as "regression to the goal", and not something I deliberately cooked up. Naive agents have just as much of a tendency to move towards the goal as experienced ones, and instead of a route memory distribution they sample from a uniform distribution (i.e. naive agents are impacted by noise instead of route memory). A priori, I did not fully appreciate that "regression to the goal" would occur, or that it would be such a driver of inter-generational improvements in efficiency.

I don't think I'm alone in this: Sasaki & Biro, and people referencing their work since, have outlined much more complex explanations for why pigeons showed (rudimentary) cumulative culture. These include ascribing more advanced social and decision making skills to pigeons.

In sum, if it indeed seems very logical that cumulative culture is baked into this simple model, it might well be that this is due to the model's breaking down of social navigation into constituent parts. Prior to this manuscript, I don't think it seemed quite so logical that regression to the goal was the reason for pigeons (and other organisms engaging in similar behaviour) to show cumulative culture.

Discussion
11. Pg 11 - Social proximity itself is not communication, especially in this context, there are no signals and no intention. Also social proximity does not have "fidelity". Maybe meant to say something like social proximity is a fundamental feature or characteristic of social species. Related to fidelity, actually there is no direct transmission mechanism coded in this model, rather information transfer emerges from the learning of waypoints while maintaining proximity, something like local enhancement. This is low-fidelity, and I guess what was intended from the sentence about fidelity.

ANSWER: The reviewer managed to distil my meaning despite my awkward articulation. The sentence has been rephrased:

"These results suggest that stepwise improvement between generations can occur when individuals simply seek proximity to others. Agents had no capacity or intent to communicate, but information transferred between them as naive followed and memorised experienced agents' routes, while their subtle goal-directed pull introduced stepwise improvement between generations." (section "Discussion")

The sentence likening social proximity to "low-fidelity" communication has been removed.

Reviewer #4:

This paper uses simulations to investigate whether cumulative cultural evolution can emerge in artificial agents that possess a minimal cognitive architecture. The model is inspired by a recent experiment that showed that pairs of homing pigeons become progressively better at solving a navigation task across successive generations. In the present model, the behaviour of artificial agents depends on 4 parameters: Goal direction, Social proximity, Route memory and Continuity (with the latter preventing erratic behaviours). The results show that route efficiency improves across generations even in the absence of sophisticated cognitive architecture.

Overall, I found the paper interesting. The main result is not entirely novel as it replicates what has been observed in the wild, but it is still nice to show that a similar process can be observed within populations composed of simple artificial agents. Moreover, the possibility of shutting down the effect of a given parameter provides interesting insights into what underlies this process. The paper lacks clarity in certain sections but with revisions it could become a valuable addition to the literature. Here are a few suggestions to improve the paper:

ANSWER: I greatly appreciate the reviewer's positive evaluation, and their constructive comments were a very welcome help in improving the manuscript.

One of my concerns is that the existing literature is not properly cited/discussed. For instance, the author suggests that it is believed that "cumulative culture relies on high-fidelity social transmission and advanced cognitive skills" (Abstract) and writes that the results demonstrate that "cumulative cultural evolution occurs even in the absence of sophisticated communication or thought" (not clear what 'thought" means here) and that "stepwise improvement between generations can occur even in the absence of high-fidelity communication". Yet, the fact that stepwise improvement between generations can occur even in the absence of high-fidelity social transmission has been demonstrated several times before (e.g. Caldwell, C. A. & Millen, A. E. Social Learning Mechanisms and Cumulative Cultural Evolution: Is Imitation Necessary? Psychol. Sci. 20, 1478-1483 (2009); Zwirner, E. & Thornton, A. Cognitive requirements of cumulative culture: teaching is useful but not essential.
Scientific Reports 5, 16781 (2015)).

ANSWER: I fully agree with the reviewer here, and in fact Zwirner & Thornton (2015) was already cited in the Discussion as an example of cumulative culture in the absence of high-fidelity social transmission. (I noted that it does require strategic social learning and advanced cognition, and I think the artificial agents offer a demonstration of the minimal cognitive architecture required for inter-generational improvements.)

That said, the reviewer is entirely correct that my writing wasn't clear enough, and didn't engage with existing literature deeply enough. On this point, I have tried to address this by introducing both referenced papers in the Introduction:

"In humans, this "ratcheting" (Tomasello et al., 1993) of socially-transmitted improvements is vital to human technological advancement (Derex, 2022), and has historically been attributed to uniquely human "high-fidelity" communication (Tennie et al., 2009). However, experimental work has shown simple emulative learning is sufficient for cumulative culture to occur (Caldwell & Millen, 2009; Zwirner & Thornton, 2015)."

and by expanding upon the point I had intended to make in the Discussion:

“To achieve this ratchet effect, agents did not require high-fidelity social communication (Tennie et al., 2009; Tomasello et al., 1993). While previous worked has demonstrated step-wise improvement between generations through emulative learning (Caldwell & Millen, 2009; Zwirner & Thornton, 2015), tasks required strategic social learning and advanced cognitive skills. The current findings outline a minimal set of cognitive abilities that is necessary and sufficient for cumulative culture to emerge.”

The author also writes that "one interpretation of [their] finding is that current definitions are too loose, and should be narrowed. An alternative conclusion is that rudimentary cumulative culture is an emergent property of systems that seek social proximity and have an imprecise memory capacity, providing a flexible complement to traditional evolutionary mechanisms". The Sasaki and Biro's paper upon which this paper is built has already triggered a debate in the literature about the former point (Mesoudi, A. & Thornton, A. What is cumulative cultural evolution? Proceedings of the Royal Society B: Biological Sciences 285, 20180712 (2018); Derex, M. Human cumulative culture and the exploitation of natural phenomena. Philosophical Transactions of the Royal Society B: Biological Sciences 377, 20200311 (2021)). The paper would serve the field better by acknowledging from the very beginning that it focuses on a minimal form of cumulative culture rather than artificially maintaining confusion (sentences such as "this process has shaped human technological innovation" are ambiguous on the evolutionary implications of the process studied here).

ANSWER: Both of these papers were already cited in the Discussion, but I agree with the reviewer that it is better to narrow down the scope of the paper in the Introduction.

One other point that puzzled me concerns the 4 parameters of the model (Goal direction, Social proximity, Route memory and Continuity). As the paper draws a parallel between this minimal form of cumulative culture and human cumulative culture, it would be useful to discuss what Continuity would correspond to in tasks other than navigation. Is that reasonable to assume that an equivalent to this parameter plays a role in human cumulative culture?

ANSWER: This is such an intriguing question! I had only really considered the technical aspects of the continuity parameter in navigation. However, the reviewer draws an interesting parallel that intuitively works: Do humans have a tendency to continue along the current trajectory, favouring to only make small and incremental changes to behaviour?

In practice, continuity is not necessary for the artificial navigators to produce inter-generational improvements. This is evident in the lesion experiments (Table 1), in which I have now included continuity in response to the reviewer’s suggestion. As a consequence, and in an attempt to stick to the Short Report format, I have not attempted to draw a parallel between agent’s continuity and a potential analogue in humans.

Also, I was surprised to see that control experiments with lesioned agents concerns all parameters but that one. What are the consequences of shutting down this parameter? If that parameter is necessary for cumulative culture does that mean that the results only hold for tasks where individuals pursue a single and fixed goal over time? (see Charbonneau, M., Strachan, J. W. A. & Winters, J. The problem with solutions: A commentary of "Blind alleys and fruitful pathways in the comparative study of cultural cognition" by Andrew Whiten. Physics of Life Reviews 44, 61-63, 11.012 (2023), for a discussion on the role of setting new goals in human cumulative culture).

ANSWER: Lesioning the continuity component is an interesting idea, and I have now implemented this. It can great reduces path efficiency (depending on w_{goal}), but inter-generational improvement in the experimental condition still occurs. The parameter is thus not necessary for cumulative culture: generational improvement occurs without it. (See Table 1 for the updated results.)

Previously, the continuity component wasn't lesioned, because I viewed it as a filler parameter and a trick to keep agents from erratic patterns. It exist primarily to reflect the physical constraints that birds have on their paths: they can't suddenly turn back upon themselves, but have to do so with a bend. The new findings that agents who have a tendency to stay their course have better efficiency than those who navigate with a noisy heading component instead.

In addition to the continuity parameter, which speaks only to individuals staying aligned with their current heading, the reviewer also brings up an important point regarding the pursuit of a fixed goal over time. Pigeons in the Sasaki & Biro study and agents in the current study both have a fixed goal. Improvements in efficiency are with respect to this fixed goal, and thus reach a ceiling when paths fully converge on the beeline. There is no expansion in the space of possible solutions. The Discussion already highlighted this:

"However, there is a limit to this process: when the direct path between start and goal is reached, no further efficiency improvements can be made."

and:

"Artificial navigators did not meet any of the extended criteria for cumulative culture, such as functional dependence, diversification into lineages, recombination across lineages, exaptation, or niche construction (Mesoudi & Thornton, 2018). In fact, their limited cognitive architecture could never achieve the expansion of the set of optimised phenomena, "Type II" cumulative cultural evolution (Derex, 2022)."

I do greatly appreciate Charbonneau's perspective, and have now used it to contextualise the above:

"However, there is a limit to this process: when the direct path between start and goal is reached, no further efficiency improvements can be made. While focussing on task efficiency offers insight into cumulative cultural evolution (Gruber et al., 2021), solutions are only one side. Humans also actively discover new problems and generalise solutions between them, which non-humans rarely do (Charbonneau et al., 2023). Artificial navigators optimise within a set of phenomena ("Type I" cumulative cultural evolution), but their limited cognitive architecture could never achieve the expansion of the set of optimised phenomena ("Type II") (Derex, 2022)."

Finally, I was wondering the extent to which the result that "Goal direction" is not necessary for cumulative culture is consistent with results showing that individuals don't need to understand what they are doing for cumulative culture to occur (e.g. Derex, M., Bonnefon, J.-F., Boyd, R. & Mesoudi, A. Causal understanding is not necessary for the improvement of culturally evolving technology. Nature Human Behaviour 3, 446-452, (2019)).

ANSWER: Due to changes made in response to another reviewer, goal-direction does seem to be necessary. (See my response to R1 for the technical explanation behind this.)

In my reading, the cited paper talks about how individuals do not need to understand the physics behind an improvement, but they do understand the goal of the task: to make the wheel cover the track as fast as possible. I think this is quite similar to artificial navigators: they know the goal, but

don't know the physics of the environment (e.g. they can't recombine landmarks into a single path). Our findings are thus quite analogous, and I have referenced the suggested paper in the Discussion.

Overall, I felt that the author does not really engage with the literature on human cumulative culture, which represents a big chunk of the literature on cumulative culture. I think that the paper would have a larger impact by better articulating its results with the existing literature.

ANSWER: The reviewer is correct that I did not engage much with the literature on human cumulative culture, and this was inspired not by lack of interest but rather by lack of space in the Short Report format.

In the revision, I have attempted to engage much more with the human literature, both in the Introduction and the Discussion.

Other comments about clarity:

a. Figure 2: Parameters are different in all conditions but only those for the experimental condition are put in the figure. This which is confusing.

ANSWER: The panels in Figure 2 show the efficiency for all three conditions (experimental, pair, and solo) under the parameters listed in the title of each panel. These parameters are the same for all three conditions. I have now clarified this in the caption:

"Parameters for the navigation model were the same in each of the three conditions, and weights are listed above each panel."

b. Can we really talk about innovation with this type of task? Maybe the term "beneficial modifications" could be used instead.

ANSWER: This term is used by Sasaki & Biro for exactly this behaviour, but I understand the reviewer's apprehension about the use of the term in this context. I have now made the terminology more explicit, by describing it as follows:

"[…] pairs in which experienced pigeons are swapped for naive ones show "innovation": beneficial modifications between generations (Sasaki & Biro, 2017)" (section "Introduction")

c. The author writes "innovations that meet core criteria for cumulative culture" but those criteria are not explicitly introduced.

ANSWER: These criteria were enumerated in the first paragraph of the Discussion, but I agree it would have been clearer to do this in the introduction. I have now moved this:

"An appealing framework describes four core criteria of cumulative cultural evolution: behaviour needs to 1) show variation introduced by interaction between individuals, 2) be passed on through social interaction, 3) improve performance, and 4) repeat over generations (Mesoudi & Thornton, 2018)." (section "Introduction")

d. Figure 2: Darker lines indicate higher levels of Wmemory. This makes the figure hard to read because lines are superimposed, which affects

ANSWER: I've reversed the mapping (lighter now indicates higher levels of w_{memory}), increased the opacity so that superimposed lines are easier to separate, and reduced the number of lines to improve clarity.

e. Figures 2 and 3: Color code changes for the Wmemory parameter between Figures 2 and 3. A consistent colour coding will help the reader.

ANSWER: Excellent suggestion; the colour schemes are now the same between Figures 2 and 3.

f. Figure S1: not clear why disks don' have the same size (does that mean that Wmemory was not varied as much for some combinations of Wgoal and Wsocial).

ANSWER: The parameter values add up to 1, so for higher values of w_{goal} and w_{social}, values of w_{memory} that would add up to over 1 are impossible. This indeed means that fewer w_{memory} options were run for some combinations of w_{goal} and w_{social}.

g. The tile seems to be poorly chosen: Why this focus on "imprecise memory"? Is that the main result of the paper?

ANSWER: I tried to keep the title short, and attempted to include the agents' goal-direction and seeking of social proximity in the phrase "social navigators". Evidently, this is not particularly clear, and thus it seemed the title focussed on the agents' memory. I have followed the reviewer's advice in changing the title to:

"Cumulative culture spontaneously emerges in artificial navigators that have goal-direction, route memory, and seek social proximity"

This captures the three necessary and sufficient components that allowed the navigators to achieve inter-generational improvement.
