# Supplementary material for: Cumulative route improvements spontaneously emerge in artificial navigators even in the absence of sophisticated communication or thought
Source: PLoS Biol. 2024 Jun 6;22(6):e3002644. doi: 10.1371/journal.pbio.3002644 (PMC11156315; doi:10.1371/journal.pbio.3002644)
Supplement: Attachment — Submitted filename: point-by-point_response.pdf [file pbio.3002644.s003.pdf]

Dear Dr Roberts,

Many thanks for your thoughts on the revised manuscript and reviewer comments. I hugely appreciate the time and care that you and your team have taken in handling this manuscript, and I am thankful to the reviewers for their constructive peer reviews. Please find a revised version attached, and a point-by-point response included below.

The main changes are the implementation of your own and the Academic Editor’s suggestions. This includes the removal of mentions of “cumulative culture” from the title and throughout the manuscript, rephrasing of the title, refocussing of the abstract, restructuring the Introduction and Discussion, and resolving outstanding reviewer comments.

I hope the current revision brings the paper up to PLOS Biology standards.

Best wishes,

Edwin

Editor

a) Please address all of the remaining requests from the reviewers.

ANSWER: Please see below for a point-by-point response of how the remaining requests from reviewers have been addressed.

b) The Academic Editor has also provided some comments, in which s/he firmly requests that you avoid the term "cumulative culture" for most of the paper, including the Title.

ANSWER: In my reading of their comments, the Academic Editor suggested changes to the study's pitch that could be conceived of as a substantial rewrite of Introduction and Discussion to avoid the area of cumulative culture, and instead focus more on animal navigation. I tried to implement as much of this as I could, while still staying true to the version of the manuscript that reviewers have seen, corrected, and (mostly) approved of.

The term "cumulative culture" has now been avoided throughout the paper, including the Title. As per Reviewer #4 and the Academic Editor's suggestions, I have also rephrased the Introduction and Discussion sections to focus on pigeon and agent behaviour.

The term "cumulative culture" is still used to introduce the background to Sasaki & Biro, and it is included in sections in the Introduction and Discussion intended to separate the current work from what readers are likely to conceive of as "cumulative culture" in humans.

The introduction now explicitly describes the aim of the study as investigating "cumulative route improvements emerged as accidental by-product during navigation in groups". The Introduction also ends on a new section titled "What is (not) cumulative culture?" in which animal vs human cumulative culture is described, and the controversy around animal vs human cumulative culture is highlighted.

The final paragraph of the Introduction is now very explicit about how agents do not meet the standard that most readers are likely to have for cumulative culture:

"*The agents employed in this study arguably do not meet the above standard. Their "innovation" is limited to an increase in efficiency, which is decidedly unlike the development of novel behaviour. While focussing on task efficiency offers insight into cumulative cultural evolution (Gruber et al., 2021), a focus on task solutions can obscure that humans also actively discover new problems and generalise solutions between them, which non-humans rarely do (Charbonneau et al., 2023). Agents also do not engage in "social learning" as it is traditionally defined: all they do is follow other individuals, without explicit demonstration or observation of a concrete task. Hence, I will refer to their outcome as "cumulative route improvements".*"

The Discussion now discusses the findings in three paragraphs that talk about cumulative improvements in minimal conditions, and not about "cumulative culture". This is followed by a clearly labelled section on whether the current findings extend to cumulative culture, which primarily separates the concept of "cumulative culture" from the cumulative improvement in the current agents.

I hope that you agree that the current manuscript is a good compromise between using material that the reviewers have seen and approved of, and not labelling the results as "cumulative culture".

c) After some discussion with my team-members, who found the current Title rather obscure, and with the Academic Editor, we suggest that you change it to something like "Cumulative route improvements spontaneously emerge in artificial navigators even in the absence of sophisticated communication or thought" (perhaps "in silico navigators" would be clearer?)

ANSWER: I struggled with finding a short title that covered the content, so I appreciate this suggestion. It has now been implemented as the new title.

d) My team-mates also found the Abstract rather difficult to comprehend, and this will need to be improved, especially for our broader readership. Specifically, two things were very unclear. Firstly, what are the "artificial agents" that you use - my understanding is that these are in silico or simulated entities, and this should be made clear in language that will be clear not only to those in your own field but also to general biologists (the majority of our readership). The second thing, which does not seem to appear in your abstract, is that you compare your model to real-life data from pigeons, finding a good fit - this should be mentioned in the Abstract, and overall the Abstract should be re-worked with the broader reader in mind.

ANSWER: Both are excellent points, and have now been incorporated. The abstract has been rewritten for clarification and to remove discussion of cumulative culture. It also specifically mentions “agent-based simulation”, and then defines “artificial agents” as “in-silico entities”. In addition, it addresses the fit to pigeon data.

e) you say in the financial declaration that you received no specific funding for this work. Can I just confirm that this is the case?

ANSWER: This is indeed the case. The project was done as part of my appointment at the University of Bristol, using existing resources, and without specific funding.

f) Many thanks for supplying the data and code. My understanding (correct me if I'm wrong) is that the data are in Zenodo, and that the code is in Github. Because Github depositions can be readily changed or deleted, please make a permanent DOI’d copy of the code (e.g. in Zenodo) and provide this URL (see below).

ANSWER: This is exactly right. I have now also included a snapshot of the code on Zenodo. This is a copy of the linked GitHub repository, so that code will persist even in the event of GitHub shutting down or the repository being deleted. (I do not foresee this happening due to my own actions, but GitHub does occasionally erroneously remove repositories, so the Editor is right to be concerned.)

g) Please cite the location of the data clearly in all relevant main and supplementary Figure legends, e.g. “The data underlying this Figure can be found in https://zenodo.org/records/XXXXXXXX" or "The data and code required to generate this Figure can be found in https://zenodo.org/records/XXXXXXXX"

ANSWER: All legends now include this statement:

The data and code required to generate this Figure can be found on Zenodo: https://doi.org/10.5281/zenodo.6944185 (data) and https://doi.org/10.5281/zenodo.10997495 (code).

As you address these items, please take this last chance to review your reference list to ensure that it is complete and correct. If you have cited papers that have been retracted, please include the rationale for doing so in the manuscript text, or remove these references and replace them with relevant current references. Any changes to the reference list should be mentioned in the cover letter that accompanies your revised manuscript.

ANSWER: The references were compiled using a reference manager, and I have ensured that it was updated on the final submission so that it accurately reflects all in-text citations and to conform to PLOS Biology's preferred referencing format. Other than formatting, no changes were made in references between the previous and the current version. To the best of my knowledge, no retracted papers were cited.

Reviewer #1

The author has revised and resubmitted their model of the cumulative cultural evolution of efficient flight paths in pigeons. I previously enjoyed reading this paper and continued to do so following its revisions. The author should be commended for their thorough improvements to the manuscript. I was particularly appreciative of their deep dive into the role of goal-direction and the results of the model now make sense (to me at least). Below I have a few minor concerns, however, overall I have no reservations in recommending this manuscript for publication.

ANSWER: Many thanks for the positive appraisal, and for pointing out the remaining issues. I have now resolved these (see below for specifics on each point).

L.54. Through the symbol for b^_other does not render correctly, appearing as an empty cube.

ANSWER: This is likely due to the publisher's PDF generation. To help that, I've now replaced all in-text references to b hat as the combination of unicode characters for b U+0302, resulting in b̂. This is the standard solution to non-standard circumflex usage (e.g. in mathematical notations), so I would expect the publisher's software to be able to render this correctly.

L55. Typo. "in components' precision" should be "in each component's precision" (note shift of apostrophe).

ANSWER: This typo is now fixed in the suggested way.

E1. It is not clear why k_memory is indexed by i. This is explained in the methods, but I would mention here it is because the precision of memory increases across flights.

ANSWER: This has now been addressed in the paragraph following the equation, by adding the following sentence:

"*Note that memory precision improves over journeys, as per evidence from pigeon flight (Mann et al., 2011).*"

**L90-98. Typos. W_goal and w_social are presented as integers (e.g. 15) instead of decimals (e.g.
0.15).**

ANSWER: Good catch! Resolved in the suggested way.

L129-150. This section is hard to follow and could be improved. Specifically:

L130-132. What is meant by "identical" is it that the same weights are used?

ANSWER: Rephrased as "[…] *employed the same weights as* [...]".

L135. Typo. ", .".

ANSWER: Typo resolved.

L136-137. Note that this necessity can be seen in that efficiency did not improve when the goal-direction system was lesioned.

ANSWER: This has now been noted, specifically by adding the following to the sentence:

"*which should not occur with lesioned goal-direction if it is dependent on regression to the goal*"

L157. In what way do these results not align with agents?

ANSWER: Now more precisely phrased as "*these weights did not align with optima for inter-generational improvement or efficiency for agents*".

L201. "demonstrates" should be "demonstrate".

ANSWER: Typo resolved.

Reviewer #2

I would like to thank the author for their clear and detailed responses, particularly to the more technical modelling queries. For my part, the rewritten article has addressed my concerns regarding the lack of background information and need for wider context, and I am happy to recommend it be published in its current form. Although, as I mentioned previously, I'm no expert in modelling, so please do defer to the more knowledgeable reviewers' opinions regarding the responses to these queries. However, I did find the author's responses to these questions (and changes made as a result) to be clear and plausible to me, and I at least was satisfied that they were addressed sufficiently.

ANSWER: Many thanks for the positive appraisal of the revision!

Reviewer #3

Many thanks to the authors for their clear responses to my comments--the manuscript is greatly improved in clarity and contribution, and I can recommend this for publication. I especially liked that the ms now highlights the mechanism as regression to the goal, and is a nice formalization of this mechanism that can lead to CCE.

I only have some lingering minor comments below, which I do not need responses to:

ANSWER: Many thanks to the reviewer for their constructive and helpful comments! Lingering issues addressed below.

L133: "instead of goal-direction, social proximity, route memory, or continuity Von Mises distributions, headings were sampled from uniform distributions"

"continuity Von Mises distributions" sounds wrong here and might confuse readers. Suggest instead to avoid it: However, headings were sampled from uniform distributions (i.e. noise) instead of being influenced by goal-direction, social proximity, route memory, or continuity.

ANSWER: Sentence rephrased as suggested.

L179: "While focussing on task efficiency offers insight into cumulative cultural evolution (Gruber et al., 2021), solutions are only one side"

I think authors meant "one-sided", although I still don't quite get what that means. Maybe meant to say something like "evolutionary paths are limited?"

ANSWER: The full stop at the end of this sentence should have been a colon, as the meaning of “one-sided” was explained in the sentence directly after:

“*Humans also actively discover new problems and generalise solutions between them, which non-humans rarely do (Charbonneau et al., 2023)*”

Resolved by combining the sentences into one, and by clarifying the meaning of the sentence. The result is as follows:

“*While focussing on task efficiency offers insight into cumulative cultural evolution (Gruber et al., 2021), a focus on task solutions can obscure that humans also actively discover new problems and generalise solutions between them, which non-humans rarely do (Charbonneau et al., 2023).*”

L203: "but adopt new sites upon population refresh"

I like the turn of phrase, but maybe something like "upon complete population replacement" would be clearer.

ANSWER: Resolved as suggested.

L205: "naive individuals conforming to efficient variants"

Would recommend "adopting efficient variants", since as I remember there was no evidence of conformity in this experiment

ANSWER: Thanks for the clarification; resolved as suggested.

L330-345: all of these reported metrics might be better off in a table, and in the supplementary. Will save you some space.

ANSWER: Thanks for the suggestion! While the reviewer is certainly correct that this would save some space in the word count, I think tables would paradoxically cost more page space than a text paragraph in a published version.

Reviewer #4

The paper has shown clear improvements, and I believe the author has appropriately addressed most of the raised comments. However, there are still some areas that need attention:

ANSWER: Many thanks for the positive appraisal of the revision, and for highlighting remaining issues. A point-by-point response is included below.

Major comments:
While the revised introduction does a better job of citing existing work, the structure remains poor, making it difficult to follow. For example, the first sentence defines cumulative culture, followed by a sentence about underlying mechanisms (line 6), then a discussion on cumulative culture in animals (lines 8-12), followed by paragraphs on alternative mechanisms/explanations (lines 13-17), and more definitions of cumulative culture later on. I recommend the author streamline the material from line 1 to 36 for greater clarity.

ANSWER: The structure has now been improved by outlining the background to Sasaki & Biro, before outlining their findings, and explaining how the current study interrogates and expands upon them. In-depth discussion of cumulative culture is now left to a separate section under a dedicated heading, to contextualise the controversies and how the current findings would not fit with readers' expectations around what "cumulative culture" is.

I think this structure is more streamlined and offers better clarity, but still holds the content requested by the reviewer and other reviewers.

The author cites the Mesoudi & Thornton definition of cumulative culture to argue that the documented phenomenon constitutes an instance of cumulative culture. However, the second criterion of this definition specifies that the behavioral variant must be passed to others via social learning. Typically, this involves a cultural demonstrator (an individual with experience) and a cultural learner (an individual naive to the task). Here, the only social mechanism is social proximity. I am not convinced this qualifies as social learning, which should be discussed further. As I mentioned in my previous review, it is crucial to articulate these results within the existing literature.

ANSWER: Mesoudi & Thornton simply note that "the behavioural variant must be passed to others via social learning" in their definitional statement, and seem to apply this relatively liberally in animal studies (see their Table S3). However, I recognise that my interpretation of their definition might be controversial, and I also appreciate the reviewer's related concern of whether the Mesoudi & Thornton definition aligns with what the wider field would consider "cumulative culture". The

Academic Editor raised similar concerns, and I have thus opted to remove mention of “cumulative culture” throughout the manuscript.

There is still discussion of cumulative culture and how the current results fit within that literature in the Introduction and the Discussion, but it is now offered under separate headings. This ensures that the content requested by reviewers is retained, but also distinguished from the current findings to better disassociate them.

I think this better articulates the results within the existing literature in the way that the reviewer envisioned.

Minor comment:
Line 135: Part of the sentence/paragraph is missing.

ANSWER: This was a typographical error (“, .” should have been “,”), and the sentence has also been improved for clarity:

“*When goal-direction was lesioned for all but the first generation, a path could be established within the first generation. This isolated goal-direction’s necessity for inter-generational improvement, which should not occur with lesioned goal-direction if it is dependent on regression to the goal.*”

COMMENTS FROM THE ACADEMIC EDITOR:

I do find, however, that the author has not sufficiently addressed reviewer 2's comment - that the paper is not in fact about cumulative culture by most people's reading of the term. Readers might be misguided by the title to think that a human-like phenomenon is under examination: the building of new technological or cultural innovations on previously existing ones. Instead it refers to a single study on gradual flight route improvements in pigeons that come about by social learning. The authors of that study have indeed argued that this qualifies as cumulative culture, but this is not widely accepted: indeed it does not contain the innovation of any novel behavioural routines (e.g. the use of a tool that spreads via social learning) - much less the building of a further unique innovation on top of another. There are more impressive examples about the gradual improvement of spatial information in animal groups - e.g. the consensus decision making in honeybee swarms (cf work by Martin Lindauer in Tom Seeley), and no one has seen the need to label these as cumulative culture. I don't think that the modelling in the present study would generalise to other cases than the particular phenomenon in pigeon navigation. So I think the author would do himself a favour if he removed "cumulative culture" from the title and instead indicated a clear focus on the specific phenomenon under investigation: route improvements in navigation via social learning. The same applies to elsewhere in the study. It's fine to discuss the study in its relation to cumulative culture, but readers will be disappointed if it's pitched as it currently is.

[...and in response to Roli Roberts' suggestion that the Title should be changed to something like "Cumulative route improvements spontaneously emerge in artificial navigators even in the absence of sophisticated communication or thought" and the whether it would be fair/appropriate to ask the author to substitute "cumulative route improvements" for "cumulative culture" throughout...:]

....yes, you have my full support for this change of the title, and the terminology throughout. I think an interesting finding has to stand on its own no matter whether one sticks a fancy label

on it or not. I think it's fine if he wants to have a brief discussion of cumulative culture somewhere, e.g. "such cumulative route improvements have even been discussed as bearing some of the elements of cumulative culture as occurs in species X, Y and Z; I suggest that my modelling might also apply to phenomenon x if the following conditions are met" or something like this.

ANSWER: The title and terminology have now been changed throughout. In addition, discussion of cumulative culture is now separated under clear headings in the Introduction and Discussion, and primarily serves to discuss the differences between animal and human cumulative culture, and to distinguish the current study from cumulative culture (i.e. to clarify how the current study is *not* cumulative culture).

This is in line with the Academic Editor’s requests, and also retains the content that reviewers have seen, requested, and approved of.
